# Construction and UAV-based inversion of integrated nitrogen diagnosis index for cotton using multispectral imagery

**DOI:** 10.3389/fpls.2026.1757798

**Published:** 2026-03-17

**Authors:** Dongdong Zhu, Qiuping Fu, Zhenghu Ma, Shudong Lin, Qinglong Geng, Ming Hong, Jinghua Zhao, Liang Ma, Yingjie Ma, Quanjiu Wang

**Affiliations:** 1 College of Water Conservancy and Civil Engineering, Xinjiang Agricultural University, Urumqi, China; 2 Xinjiang Future Irrigation District Engineering Technology Research Center, Urumqi, China; 3 Institute of Agricultural Resources and Environment, Xinjiang Academy of Agricultural Sciences, Urumqi, China; 4 National Key Laboratory of Water Engineering Ecological Environment in Arid Areas, Xi’an University of Technology, Xi’an, China

**Keywords:** cotton, integrated nitrogen diagnosis index, machine learning, nitrogen diagnosis, nitrogen nutrition index, UAV

## Abstract

**Introduction:**

Accurate and stable diagnosis of cotton nitrogen status across growth stages is essential for precision fertilization in drip-irrigated systems. However, the instability of conventional nitrogen-related indicators across different phenological stages often reduces diagnostic performance and limits their broader application.

**Methods:**

A field experiment was conducted in Xinjiang, China, under four irrigation levels (60%, 80%, 100%, and 120% ET_c_) and four nitrogen application rates (0, 245, 300, and 350 kg N ha^-1^). UAV multispectral imagery was acquired at the squaring, flowering, boll-setting, and boll-opening stages. Based on ground-measured leaf area index (LAI) and upper-canopy leaf nitrogen weight (LNWupper), an Integrated Nitrogen Diagnosis Index (INDI) was developed. Random Forest (RF), Gradient Boosting Decision Tree (GBDT), and Extreme Gradient Boosting (XGBoost) models were used to evaluate the inversion performance of INDI. In addition, the nitrogen nutrition index (NNI), derived from the critical nitrogen dilution curve, was used to validate the diagnostic stability of INDI.

**Results:**

Multispectral vegetation indices were strongly correlated with LAI, LNWupper, and INDI, with red-edge- and near-infrared-based indices showing the highest sensitivity. Among the three models, XGBoost achieved the best inversion accuracy for INDI (R^2^ = 0.85, RMSE = 0.61). INDI was significantly correlated with NNI across growth stages, with R^2^ values of 0.58, 0.77, 0.81, and 0.70 at the squaring, flowering, boll-setting, and boll-opening stages, respectively, and the highest accuracy observed at the boll-setting stage. Moreover, the spatial distribution maps of INDI effectively distinguished nitrogen differences under different water–nitrogen treatments and were consistent with NNI-based classifications.

**Discussion:**

INDI accurately captured nitrogen dynamics throughout cotton growth, and the INDI–XGBoost framework provided a robust approach for high-precision spatial nitrogen diagnosis. These results support precision fertilization management in drip-irrigated cotton fields in Xinjiang.

## 1 Introduction

Cotton is one of the most important commercial crops worldwide, and its yield and fiber quality are highly dependent on nitrogen supply (Gitelson and Merzlyak, 1994). Nitrogen plays critical roles in chlorophyll synthesis, photosynthesis, and dry matter accumulation, and strongly influences boll formation, boll weight, and final yield (Clevers and Gitelson, 2013). In drip-irrigated cotton systems in Xinjiang, water and nitrogen management are highly coupled due to frequent irrigation events and substantial nitrogen movement with irrigation water. As a result, nitrogen-use efficiency remains low, and excessive nitrogen application and environmental losses are still common (Haboudane et al., 2004). Therefore, developing a rapid, stable, and growth-stage-independent nitrogen diagnosis method is essential for precision fertilization and sustainable agricultural production.

In recent years, unmanned aerial vehicle (UAV) remote sensing has shown great potential in crop nitrogen monitoring due to its high temporal–spatial resolution and operational flexibility (Ulrich, 1952; Wu et al., 2008). Multispectral imagery captures canopy structure, pigment concentration, and physiological status, with red-edge and near-infrared bands being particularly sensitive to nitrogen variation (Lemaire et al., 2008; Gastal et al., 2015b). Numerous studies have demonstrated that vegetation indices such as NDVI, NDRE, and MRETVI derived from multispectral imagery are significantly correlated with chlorophyll content, nitrogen concentration, LAI, and other physiological traits, enabling nondestructive estimation of crop nitrogen status (Lemaire and Gastal, 1997; Inoue et al., 2012b).However, current nitrogen-retrieval research still faces two major limitations:(1) Dependence on single growth stages or single physiological indicators, which reduces diagnostic robustness and prevents full-season applicability. For example, SPAD is sensitive to light conditions and leaf position, LAI is easily affected by canopy structural variability, and leaf nitrogen concentration is strongly influenced by the dilution effect, making cross-stage comparison difficult (Bradstreet, 1954). (2) Large spectral differences among growth periods, which reduces the generalization ability of inversion models across phenological stages.

Upper-canopy leaf nitrogen weight (LNWupper) provides a direct measure of nitrogen accumulation in functional leaves, whereas leaf area index (LAI) describes canopy structure and photosynthetic capacity. Combining these two indicators may overcome the limitations of single physiological metrics. However, research integrating LAI and LNWupper into a unified nitrogen diagnostic index remains scarce. Meanwhile, machine learning algorithms—such as random forest (RF), gradient boosting decision tree (GBDT), and extreme gradient boosting (XGBoost)—have shown strong capabilities for modeling nonlinear spectral–physiological relationships, especially in high-dimensional feature environments (Lemaire et al., 2007).

To address these issues, this study constructs an Integrated Nitrogen Diagnosis Index (INDI) by integrating LAI and LNWupper and combines the index with UAV multispectral imagery and machine learning to achieve stable nitrogen diagnosis across multiple cotton growth stages. The specific objectives of this study are to: (1) analyze the relationships among LAI, LNWupper, and multispectral vegetation indices; (2) develop a growth-stage-independent INDI and assess its inversion performance using RF, GBDT, and XGBoost; (3) validate the diagnostic capability of INDI during four key growth stages using the nitrogen nutrition index (NNI) derived from critical nitrogen dilution curves. This study provides a novel, stable, and spatially explicit nitrogen diagnostic method that supports precision nitrogen management in drip-irrigated cotton fields in Xinjiang.

## 2 Materials and methods

### 2.1 Study area

**Figure 1:**
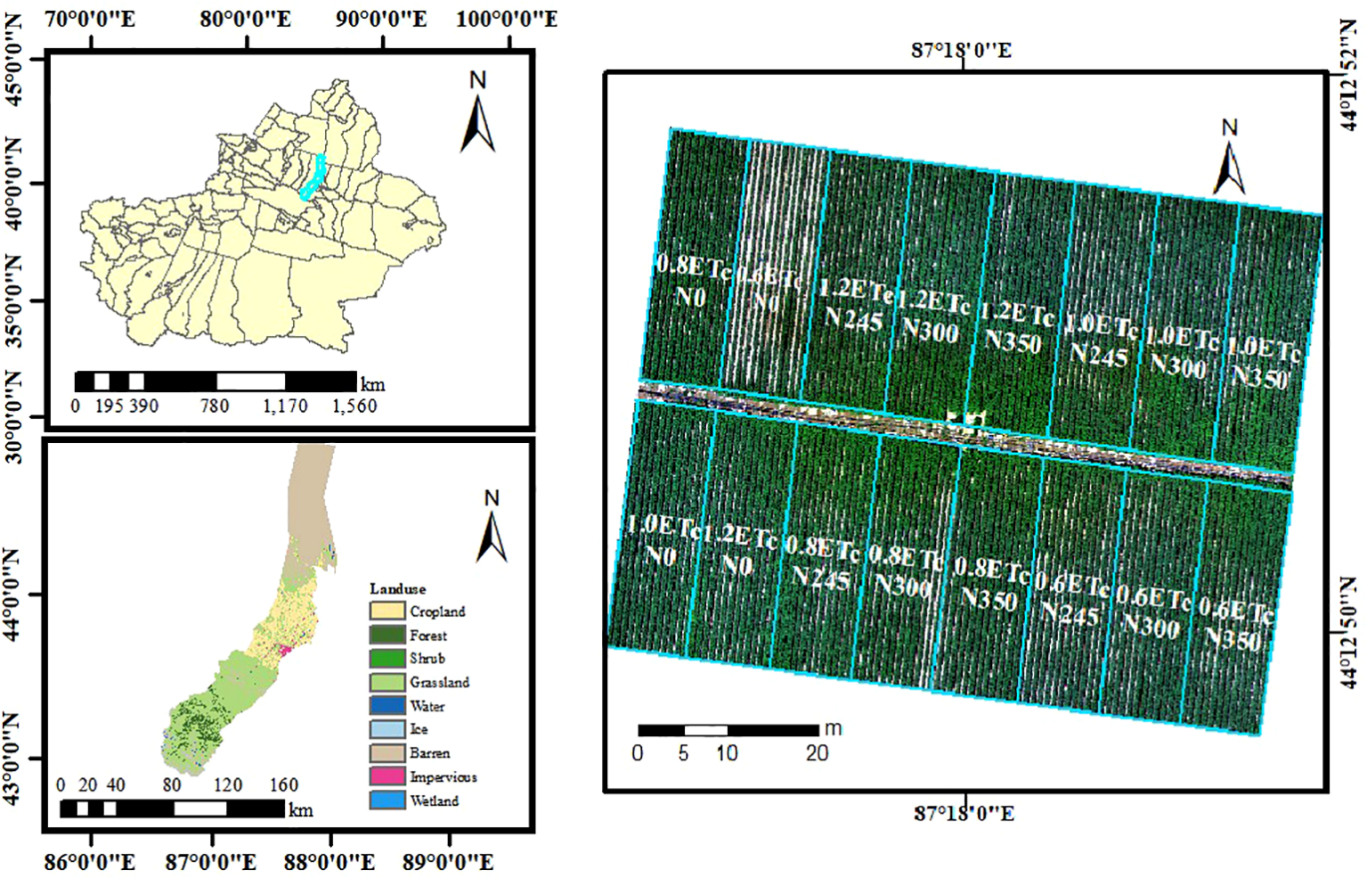
Location of the study area and layout of the experimental plots.

The field experiment was conducted from April to October 2025 at Huaxing Farm, Daxiqu Town, Changji Hui Autonomous Prefecture, Xinjiang, China (87°30′38″E, 44°21′59″N) (**Figure 1**). The region has a typical continental arid climate, with an annual sunshine duration of 2700 h, mean annual temperature of 6.8 °C, frost-free period of 160–190 days, and annual precipitation of approximately 190 mm. The soil is classified as clay loam, containing 12.90 g kg^−^¹ organic matter, 35.65 mg kg^−^¹ available phosphorus, 479.5 mg kg^−^¹ available potassium, and 0.67 g kg^−^¹ total nitrogen. Soil bulk density is 1.49 g cm^−^³, with groundwater depth below 10 m and a field capacity of 22%.Groundwater with mineralization below 1.0 g L^−^¹ served as the irrigation source. A micro-meteorological station was installed at the site to record daily air temperature, precipitation, relative humidity, and other environmental variables.

### 2.2 Experimental design

Two factors—irrigation and nitrogen application—were included in the experiment. Irrigation was applied every seven days at four levels: 60% ET_c_, 80% ET_c_, 100% ET_c_, and 120% ET_c_, where ET_c_refers to crop evapotranspiration. Nitrogen fertilizer was applied at four levels: 0, 245, 300, and 350 kg N ha^−^¹, resulting in a total of 16 treatments (**Table 1**). Phosphorus and potassium fertilizers were applied following local agronomic practices at 150 kg·ha^−^¹ each. Crop evapotranspiration was estimated using Equation 1.

**Table 1:** Experimental treatments and irrigation–fertilization levels.

Treatment	Irrigation amount (mm)	Nitrogen application rate (kg N ha^−^¹)
W0.6N0	60%ETc	0
W0.6N245		245
W0.6N300		300
W0.6N350		350
W0.8N0	80%ETc	0
W0.8N245		245
W0.8N300		300
W0.8N350		350
W1.0N0	100%ETc	0
W1.0N245		245
W1.0N300		300
W1.0N350		350
W1.2N0	120%ETc	0
W1.2N245		245
W1.2N300		300
W1.2N350		350

(1) $$ET_{c}=ET_{0}\times K_{c}$$

where ET_c_ is crop water requirement (mm), and Kc is the crop coefficient. According to FAO recommendations, Kc values for cotton were 0.70 (initial stage), 1.15 (mid-season), and 0.75 (late season). The reference evapotranspiration (ET_0_) was calculated using Equation 2 (Hou, 2022).

(2) $$ET_{o}=\frac{0.408\Delta (R_{n}-G)+\gamma \frac{900}{T+273}u_{2}(e_{s}-e_{a})}{\Delta +\gamma (1+0.34u_{2})}$$

Cotton variety “Zhongmian 113” was sown on 23 April 2025, topped on 21 July, and harvested on 26 September. The field followed a “one-film, three-tape, six-row” machine-picking configuration with row spacing of 10 + 66 + 10 cm and a plant spacing of 8.5 cm. Drip tapes were placed in the center of the narrow rows, with an emitter flow rate of 2.6 L·h^−^¹ and emitter spacing of 25 cm. Except for the emergence irrigation and the final irrigation event, fertilizers were delivered through drip irrigation. Each plot was equipped with a water meter for precise irrigation control. Nitrogen fertilizer was applied as urea (N ≥ 46%), phosphorus as monoammonium phosphate (P_2_O_5_ ≥ 46%), and potassium as potassium sulfate (K_2_O ≥ 50%).

### 2.3 UAV multispectral image acquisition and preprocessing

To minimize the effects of shadow and plant movement, UAV image acquisition was performed under clear-sky conditions with wind speeds <3 m s^−^¹ between 11:00 and 14:00. A Matrice 350 RTK UAV (DJI, China) equipped with a RedEdge-P multispectral camera collected imagery at four growth stages. The sensor contains five narrow bands (**Table 2**) suitable for vegetation parameter retrieval. Flights were conducted at 30 m altitude with 80% forward and 75% side overlap. Radiometric calibration was performed before and after each flight using calibrated reflectance panels. Due to atmospheric variability and inherent sensor distortions, the initial images required preprocessing (Bansal et al., 2009). Radiometric correction, band alignment, and mosaicking were performed using Pix4D, followed by geometric correction and clipping in ArcGIS 10.7. High-accuracy RTK ground control points were used to ensure precise geometric alignment across bands. All images were clipped to the experimental boundaries to maintain consistent spatial extent and pixel alignment.

**Table 2:** Parameters of the multispectral camera and reflectance of the gray reference panel.

Band No.	Band name	Central wavelength (nm)	Bandwidth/mm	Gray panel reflectance/%
1	Blue	475	32	65
2	Green	560	27	63
3	Red	668	14	62
4	Red-edge	717	12	61
5	NIR	842	57	60

### 2.4 Measurement of leaf area index

At each growth stage, leaf area index (LAI) was measured using destructive sampling. Nine plants were randomly selected within each treatment, and LAI was determined using the punching-weighing method described by Tao & Lin (Tao and Lin, 2006). LAI was calculated using Equation 3.

(3) $$LAI=\frac{\text{the leaf area }}{\text{the ground area occupied }}$$

Where the leaf area is the total leaf area estimated from punched samples and the ground area occupied is the punched leaf disc area. The average LAI of the nine plants represented the treatment-level LAI.

### 2.5 Leaf nitrogen weight measurement

Leaf samples were oven-dried, ground, and passed through a 1-mm sieve. Subsamples were digested using H_2_SO_4_–H_2_O_2_ and analyzed for nitrogen concentration (LNC, %) using the Kjeldahl method (Inoue et al., 2012a).

Leaf nitrogen weight (LNWupper), representing nitrogen accumulation per unit leaf area, was calculated following Sokal & Braumann (Sokal and Braumann, 1980). LNWupper was calculated using Equation 4.

(4) $$LNW_{upper}=LDW_{upper}\times LNC_{upper}$$

Where LDWupper is the leaf dry weight per unit area (g m^−^²), and LNCupper is nitrogen concentration of upper-canopy leaves(%).

### 2.6 Construction of the integrated nitrogen diagnosis index

To achieve a robust, growth-stage-independent diagnosis of cotton nitrogen status, we constructed an Integrated Nitrogen Diagnosis Index (INDI) by integrating leaf area index (LAI) and upper-canopy leaf nitrogen weight (LNWupper). Because LAI and LNWupper differ in units, magnitude, and stage-dependent responses, a direct combination may lead to scale dominance and reduced comparability across stages. Therefore, both indicators were standardized prior to integration. The coefficient of variation (CV) was subsequently calculated to quantify their relative dispersion across treatments and growth stages.

#### 2.6.1 CV-based weighting

We used the coefficient of variation (CV) to quantify the relative dispersion of LAI and LNWupper under multiple treatments and growth stages. CV is dimensionless (CV = SD/mean) and thus allows direct comparison of variability between indicators with different units and scales. Based on the principle that indicators with lower relative variability should contribute more to a stable integrated index, we adopted an inverse-CV weighting scheme (1/CV) and normalized the weights to reduce the dominance of highly variable indicators and improve cross-stage robustness.

For each indicator *i* (where *i* = 1 denotes LAI and *i* = 2 denotes ${\text{LNW}}_{\text{upper}}$), CV was calculated as (Gastal et al., 2015a). The coefficient of variation (CV) was calculated using Equation 5.

(5) $$CV_{i}=\frac{\sigma_{i}}{\mu_{i}}$$

where ${\text{μ}}_{\text{i}}$ and ${\text{σ}}_{\text{i}}$ are the mean and standard deviation of indicator i, respectively. The corresponding weight ${\text{w}}_{\text{i}}$ was computed by inverse-CV weighting and normalized as. The normalized inverse-CV weight was calculated using Equation 6.

(6) $$w_{i}=\frac{{\left(CV_{i}\right)}^{-1}}{\sum_{k=1}^{n}{\left(CV_{k}\right)}^{-1}},\;n=2$$

#### 2.6.2 Stage-wise standardization and INDI formulation

To remove scale differences among growth stages, LAI and LNWupper were Z-score standardized within each growth stage (stage-wise standardization) prior to integration. Stage-wise standardization was performed using Equation 7.

(7) $$Z(X_{i})=\frac{X_{i}-\mu_{i}}{\sigma_{i}}$$

Finally, INDI was defined as the weighted linear combination of the standardized indicators. INDI was calculated using Equation 8.

(8) $$INDI=\sum_{i=1}^{n}w_{i}\cdot Z(X_{i})$$

#### 2.6.3 Comparison with alternative weighting methods

To verify that the weighting choice is not arbitrary, we compared the inverse-CV weighting with equal weighting and entropy weighting. The resulting weights are summarized in **Table 3** (detailed calculations are provided in **Supplementary Table 1**). Based on our dataset, the inverse-CV weights were w_LAI_=0.6000 andw_LNW_=0.4000, while the entropy weights were w_LAI_=0.3266 and w_LNW_=0.6734. Moreover, INDI values derived from different weighting schemes showed high agreement across growth stages (Spearman’s ρ = 0.87–0.99; **Supplementary Table 2**), indicating that our main conclusions are robust to the weighting strategy. A visual comparison of the weighting schemes is provided in **Supplementary Figure 1**.

**Table 3:** Comparison of weighting schemes for constructing INDI based on LAI and LNWupperz.

Weighting method	W _LAI_	W _LNWupper_
CV-based (1/CV)	0.60	0.40
Equal weighting	0.50	0.50
Entropy weighting	0.33	0.67

### 2.7 Nitrogen nutrition index

The concept of critical nitrogen concentration (Nc) was introduced by Ulrich (Gitelson et al., 2003a), representing the minimum nitrogen required to achieve maximum biomass. Nc follows a dilution pattern with biomass. The critical nitrogen concentration was calculated using Equation 9.

(9) $$Nc=a\times DM_{max}^{-b}$$

where a is the nitrogen concentration at 1 Mg ha^−^¹ dry matter, b is the dilution coefficient, and DM is aboveground dry matter.

The nitrogen nutrition index (NNI), widely used for nitrogen diagnosis (Clevers and Gitelson, 2013), was calculated using Equation 10.

(10) $$NNI=\frac{N_{i}}{N_{c}}$$

Where NNI = 1 indicates optimal nitrogen status; NNI < 1 indicates nitrogen deficiency; NNI > 1 indicates nitrogen surplus.

### 2.8 Selection of vegetation indices

Vegetation indices (VIs) are calculated by combining reflectance values from different spectral bands, which can enhance the spectral responses of canopy structure, chlorophyll content, and nitrogen-related physiological characteristics. They are important tools for remotely retrieving crop growth and nutritional status. Numerous studies have shown that the red-edge band and near-infrared (NIR) region are the most sensitive to changes in vegetation nitrogen and chlorophyll content, and can effectively alleviate saturation effects in the visible region; therefore, they are widely used in agricultural nitrogen diagnosis (Delegido et al., 2011; Wu et al., 2020). In contrast, traditional visible-light indices (such as NDVI and EVI) are prone to saturation under medium-to-high canopy coverage conditions, which reduces their sensitivity to nitrogen variation (Haboudane et al., 2004; Gitelson, 2005). Based on the spectral characteristics of the multispectral sensor, the nitrogen absorption mechanisms of cotton leaves and canopy, and previous studies on nitrogen inversion, 14 representative vegetation indices were selected from five major categories—visible-light indices, structural indices, red-edge indices, soil-adjusted indices, and composite indices (**Table 4**). The final inputs were explicitly reported for reproducibility: LAI (MRETVI, RVI, RERVI), LNWupper (NGBDI, NGI, NDRE), and INDI (MRETVI, RERVI, NLI, EVI).

**Table 4:** Vegetation index and calculation formula.

Note	Vegetation Index	Formula
1	MRETVI (Qi et al., 1994)	1.2*[1.2*(NIR – Green) – 2.5*(Red – Green)]
2	RVI (Gitelson et al., 1996)	NIR/Red
3	RERVI (Gitelson and Merzlyak, 1996)	NIR/RE
4	NGI (Barnes et al., 2000)	(Green – Red)/(Green + Red)
5	NPCI (Goel, 1988)	(Red – Blue)/(Red + Blue)
6	NDRE (Gitelson, 1996)	(NIR – RE)/(NIR + RE)
7	NLI (Huete, 1988)	(NIR² – Red)/(NIR² + Red)
8	VARI (Yao et al., 2020)	(Green – Red)/(Green + Red – Blue)
9	NDVI (Li et al., 2022)	(NIR – Red)/(NIR + Red)
10	EVI (Gitelson et al., 2003b)	2.5*(NIR – Red)/(NIR + 6*Red – 7.5Blue + 1)
11	NGBDI (Liu et al., 2021)	(Green – Blue)/(Green + Blue)
12	OSAVI (Chen and Guestrin, 2016)	(NIR – Red)/(NIR + Red + 0.16)
13	RENDVI (Mistele and Schmidhalter, 2008)	(RE – Red)/(RE + Red)
14	SAVI (Zhu et al., 2018)	1.5*(NIR – Red)/(NIR + Red + 0.5)

### 2.9 Model construction and validation

To achieve nitrogen indicator inversion of cotton based on UAV multispectral data, vegetation indices (VIs) extracted from each growth stage were used as input variables, and the Integrated Nitrogen Diagnosis Index (INDI) was used as the response variable. Three machine learning models—Random Forest (RF), Gradient Boosting Decision Tree (GBDT), and Extreme Gradient Boosting (XGBoost)—were constructed. All models were trained and validated in Python using the Scikit-learn and XGBoost libraries.Before modeling, variables were standardized, and Pearson correlation matrices and variance inflation factors (VIF) were used to evaluate multicollinearity. When the correlation coefficient between indices was |r| > 0.90 or VIF > 10, redundant variables were removed to reduce the effect of high-dimensional multicollinearity on model stability. This study conducted feature screening separately for the three prediction targets (LAI, LNWupper and INDI) to ensure that the selected inputs are target-specific and stable across growth stages.

A total of 48 samples were collected at each growth stage, with 240 samples collected overall. Seventy percent of the samples were used for model calibration and the remaining 30% for validation. Five-fold cross-validation was used to optimize hyperparameters, including the number of trees, maximum depth, learning rate, and minimum number of samples for node splitting, to improve model generality and avoid overfitting. Model performance was comprehensively evaluated using the coefficient of determination (R²), root mean square error (RMSE), and relative error (RE). R² reflects the model’s explanatory power for INDI variation, RMSE represents the absolute deviation of prediction error, and scatter plots of predicted vs. observed values were generated for both the training and validation sets. Model performance was evaluated using Equations 11–13.

(11) $$R^{2}=1-\frac{\sum_{i=1}^{n}{\left(y_{i}-{\hat{y}}_{i}\right)}^{2}}{\sum_{i=1}^{n}{\left(y_{i}-{\bar{y}}_{i}\right)}^{2}}$$

(12) $$RMSE=\sqrt{\frac{1}{n}\sum_{i=1}^{n}{\left({\hat{y}}_{i}-y_{i}\right)}^{2}}$$

(13) $$RE=\frac{1}{n}\sum_{i=1}^{n}\frac{\left|{\hat{y}}_{i}-y_{i}\right|}{y_{i}}\times 100\%$$

where ${\text{y}}_{\text{i}}$ is the observed value, ${\hat{\text{y}}}_{\text{i}}$ is the predicted value, and ${\bar{\text{y}}}_{\text{i}}$ is the mean of the observed values of the dataset; n is the number of observations, and i is the index of summation in increments of 1.

Using the optimal set of model parameters, the inversion model was applied to the UAV multispectral orthomosaic to generate the spatial distribution map of INDI, which was subsequently classified into three nitrogen-status levels—nitrogen deficient, nitrogen adequate, and nitrogen excessive—according to the nitrogen diagnostic criteria.

## 3 Results and analysis

### 3.1 Correlation analysis between cotton nitrogen diagnosis indices and common vegetation indices

**Figure 2:**
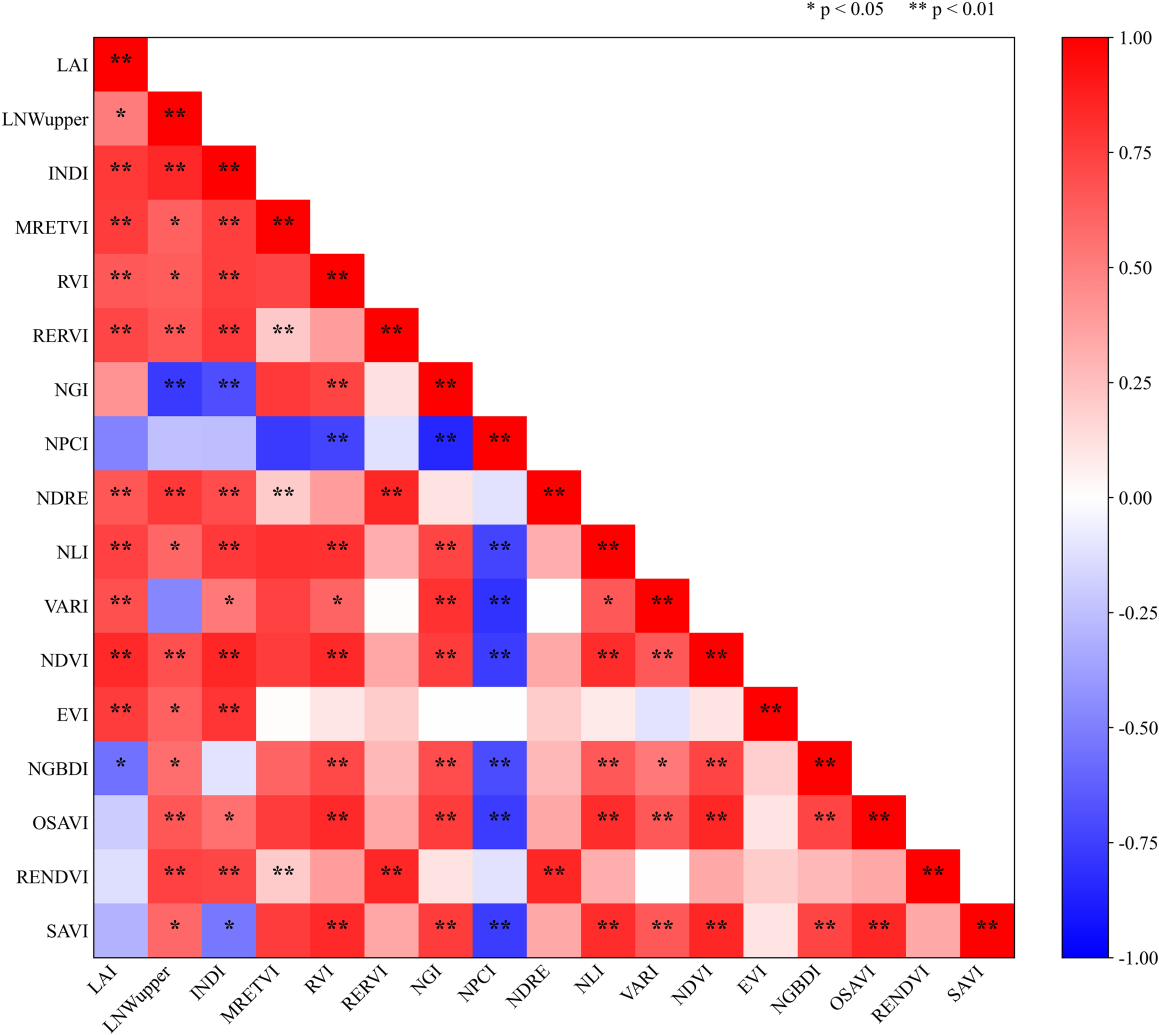
Correlation analysis between cotton nitrogen diagnosis indices and commonly used vegetation indices.

Correlation analyses were performed between LAI, upper-canopy leaf nitrogen weight (LNWupper), and the Integrated Nitrogen Diagnosis Index (INDI) across different cotton growth stages and a set of commonly used vegetation indices, as shown in **Figure 2**. The results indicated that LAI exhibited relatively high and positive correlations with MRETVI, RVI, and RERVI. The absolute correlation coefficients between LNWupper and NGBDI, NGI, and NDRE ranged from 0.6 to 0.8, with NGI and NDRE showing absolute correlation coefficients exceeding 0.75 and demonstrating highly significant relationships. INDI also exhibited strong correlations with vegetation indices such as MRETVI,RERVI, NLI, and EVI. These patterns are consistent with canopy optical mechanisms. LAI primarily represents canopy structure and effective leaf area and thus tends to align with NIR-dominated indices (e.g., RVI/RERVI) and vigor-related formulations (e.g., MRETVI) that respond strongly to canopy scattering. In contrast, LNWupper more directly reflects upper-canopy nitrogen/chlorophyll status; nitrogen-induced changes typically strengthen red absorption and modify red-edge characteristics, explaining the stronger associations with indices involving visible absorption and the red-edge region (e.g., NGI and NDRE). Because INDI integrates LAI (structural capacity) and LNWupper (upper-canopy nitrogen status), it bridges structural and biochemical signals and therefore correlates well with multiple indices (e.g., MRETVI, RERVI, NLI, and EVI). It should be noted that pooling samples across growth stages may partially dilute stage-specific sensitivities due to changes in canopy closure, soil-background influence, and pigment dynamics. This motivates the subsequent modeling strategy aiming for robust performance under multi-stage heterogeneity.

### 3.2 Construction of cotton nitrogen diagnosis models based on machine learning algorithms

**Figure 3:**
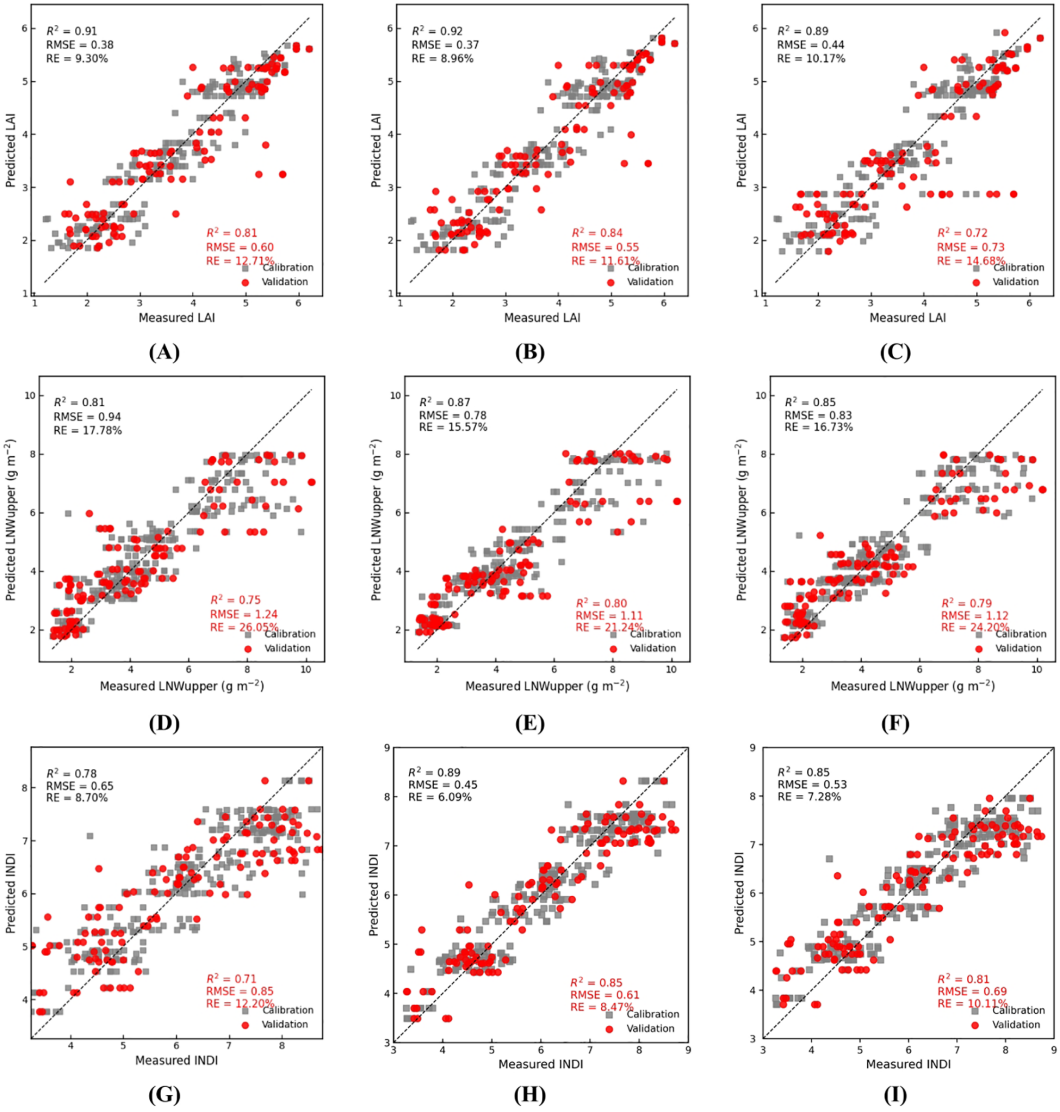
Training and validation results of machine learning algorithms: **(A, D, G)** represent the RF model; **(B, E, H)** represent the XGB model; and **(C, F, I)** represent the GBDT model.

Model training and evaluation were conducted on the pooled all-stage dataset to assess robustness and generalization under multi-stage heterogeneity. Three machine learning algorithms—Random Forest (RF), Gradient Boosting Decision Tree (GBDT), and Extreme Gradient Boosting (XGBoost)—were used to construct cotton nitrogen diagnosis models, and the prediction accuracy of each model was compared. Based on the correlation analysis, vegetation indices with absolute correlation coefficients greater than 0.6 with the target variables (LAI, LNWupper, and INDI) were selected as model input variables. A total of 240 samples of LAI, LNWupper, and INDI collected across different growth stages were divided into training and validation sets at a 70% and 30% ratio, respectively. Five-fold cross-validation was applied during model training to enhance robustness, reduce overfitting, and improve model generalization. The modeling and validation results are presented in **Table 5** and **Figure 3**.

**Table 5:** Comparison of model prediction accuracy.

Model variable	RF		XGB		GBDT	
	R^2^	RMSE	R^2^	RMSE	R^2^	RMSE
LAI	0.81	0.6	0.84	0.55	0.72	0.73
LNWupper	0.75	1.24	0.80	1.11	0.79	1.12
INDI	0.71	0.85	0.85	0.61	0.81	0.69

For LAI, all three models showed good fitting performance, with the XGB model achieving the highest accuracy, indicating its stronger capability in capturing nonlinear characteristics of LAI. For LNWupper prediction, the XGB model again performed best (R² = 0.80, RMSE = 1.11), followed by the GBDT model (R² = 0.79, RMSE = 1.12), while the RF model performed relatively poorly (R² = 0.75, RMSE = 1.24).The overall RMSE of LNWupper was higher than that of LAI, reflecting the greater biological variability inherent in nitrogen accumulation, which presents higher challenges for prediction accuracy. For INDI, the XGB model again achieved the best performance (R² = 0.85, RMSE = 0.61), and all three models showed higher prediction accuracy for INDI than for LAI and LNWupper. Under pooled multi-stage conditions, the relationships between vegetation indices and target traits are inherently nonlinear and interaction-driven due to changes in canopy closure, soil-background contribution, pigment dynamics, and nitrogen dilution processes across stages. XGBoost (gradient-boosted decision trees) can flexibly capture nonlinear response curves and high-order feature interactions, while regularization and ensemble boosting typically improve generalization in noisy and heterogeneous datasets. In contrast, linear models may underfit stage-dependent nonlinearities, and simpler single-tree approaches can be less stable when predictors are correlated. The selected indices jointly represent complementary information. NIR-driven ratio/vigor indices (e.g., RVI, RERVI, and MRETVI) primarily track canopy structural scattering and effective leaf area, which relates closely to LAI. Indices involving visible absorption and/or the red-edge region (e.g., NGI, NGBDI, NDRE, and EVI) are more sensitive to chlorophyll/nitrogen-related biochemical variation, supporting the prediction of LNWupper. NLI further contributes robustness to brightness/background variability. Combining these indices therefore provides both structural and biochemical cues, which is beneficial for stable inversion of INDI under pooled multi-stage conditions.

The high predictability of INDI indicates that this index integrates both canopy structural and nitrogen-related physiological characteristics, enabling the models to more stably and accurately capture changes in nitrogen status. Overall, the XGB model achieved the highest R² and lowest RMSE across all three target variables, outperforming both RF and GBDT. Therefore, XGB was selected as the optimal model for subsequent spatial inversion of INDI and nitrogen diagnosis.

### 3.3 Multispectral image inversion based on INDI

**Figure 4:**
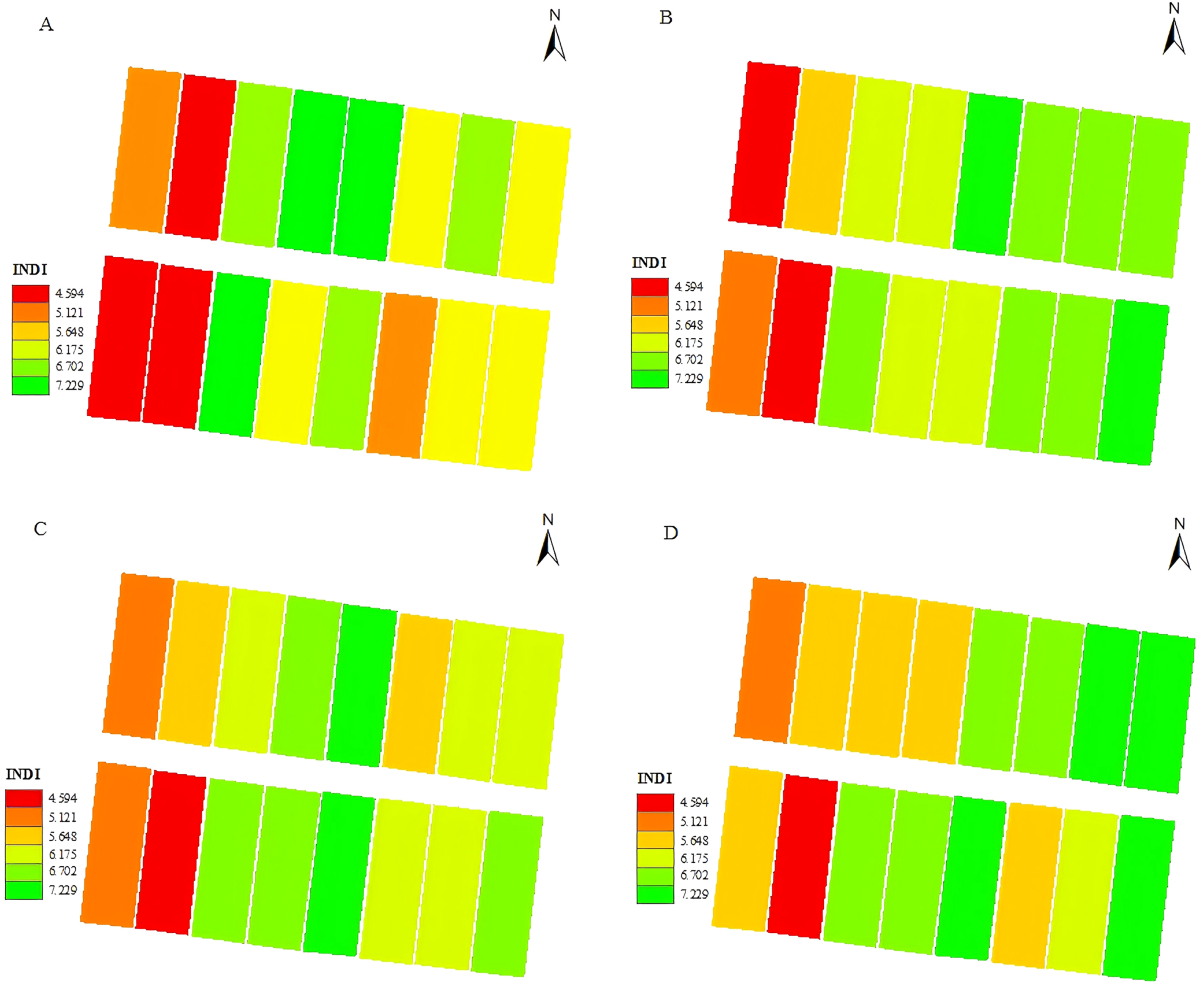
Spatial distribution of INDI at different cotton growth stages. **(A-D)** correspond to the INDI spatial distribution at the squaring, flowering, boll-setting, and boll-opening stages, respectively.

Based on the machine learning model training results, the best-performing XGBoost model (R² = 0.85, RMSE = 0.61) was selected to perform pixel-level inversion of the multispectral imagery, generating INDI spatial distribution maps of cotton fields at different growth stages (**Figure 4**). The analysis showed that under treatments with higher water and nitrogen supply—W1.0N300, W1.0N350, W1.2N300, and W1.2N350—cotton exhibited favorable nitrogen nutritional status, while in the N0 treatment, cotton exhibited nitrogen deficiency, indicating the need for timely nitrogen supplementation. This further demonstrates that INDI, as a composite diagnostic index integrating canopy structure and leaf nitrogen information, can effectively reveal nitrogen distribution patterns and spatial variability within the field.

### 3.4 Validation of INDI inversion results from multispectral imagery

**Figure 5:**
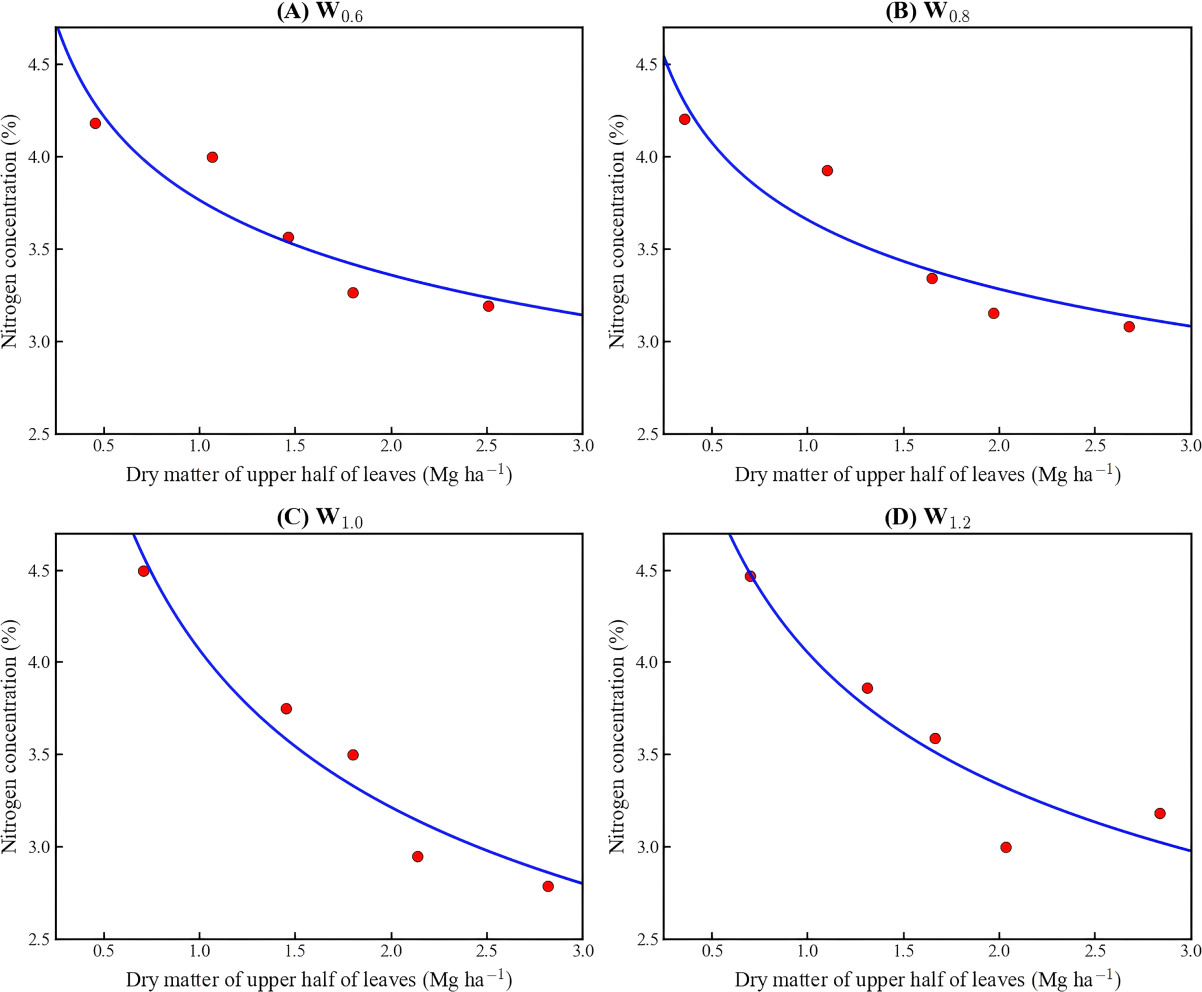
Critical nitrogen dilution curves of upper-canopy leaves under different irrigation levels. **(A-D)** represent irrigation levels of 60% ETc, 80% ETc, 100% ETc, and 120% ETc, respectively.

Following the method proposed by Ulrich, critical nitrogen dilution curves based on upper-canopy leaves were established for the four irrigation treatments, as shown in **Figure 5**. The results showed that nitrogen concentration gradually decreased over time.

The model parameters are shown in **Table 6**. As presented in the table, both parameters a and b increased with increasing irrigation amount. By fitting a and b against their corresponding irrigation levels, the relationships between irrigation amount and parameters a and b were determined. The fitted relationship between irrigation amount and parameter a is shown in Equation 14.

**Table 6:** Model parameters for the critical nitrogen concentration dilution curve for cotton.

Treatment	Irrigation amount (mm)	a	b	R²
W_0.6_	243.19	2.28×10^−^²	0.38	0.88
W_0.8_	324.26	2.41×10^−^²	0.40	0.91
W_1.0_	405.32	2.55×10^−^²	0.42	0.90
W_1.2_	486.38	2.61×10^−^²	0.43	0.88

(14) $$a(I)=9.41\times 10^{-8}I^{\;2}-5.76\times 10^{-5}I+0.0463,\;R^{2}=0.99$$

The fitted relationship between irrigation amount and parameter b is shown in Equation 15.

(15) $$b(I)=-3.50\times 10^{-6}I^{\;2}+2.96\times 10^{-3}I-0.3732,\;R^{2}=0.98$$

The critical nitrogen concentration (Nc) dilution curves for cotton under different irrigation levels was expressed by Equation 16:

(16) Nc=(9.41×10-8I 2−5.76×10-5I+0.0463)×DM−(−3.50×10−6I 2+2.96×10−3I−0.3732)

**Figure 6:**
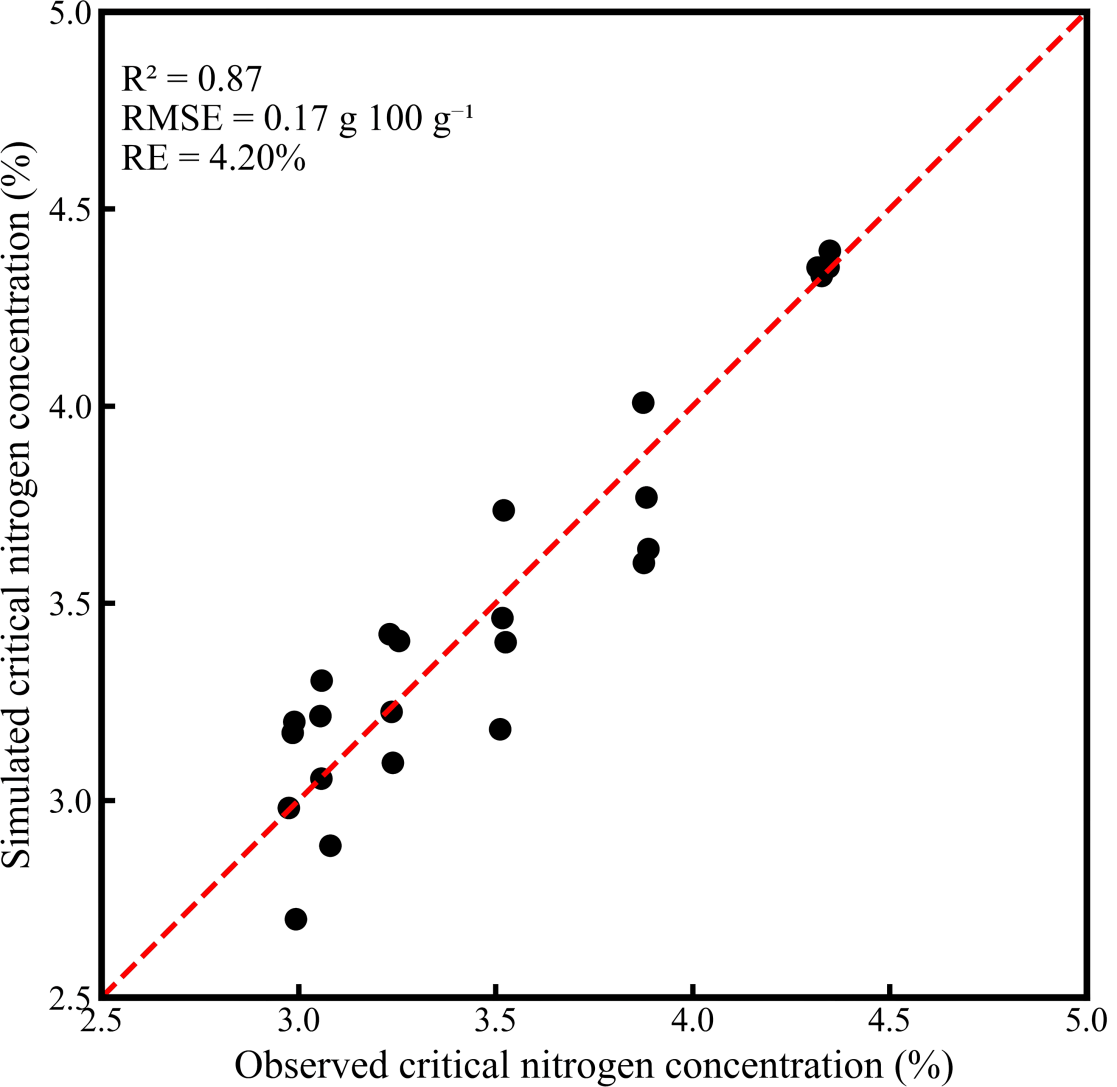
Comparison of simulated and observed critical nitrogen concentrations.

By substituting irrigation levels and aboveground dry matter of upper-canopy leaves into the equation, simulated Nc values were obtained. The relationship between simulated and observed Nc values is illustrated in **Figure 6**. The results indicated an R² of 0.87, an RMSE of 0.17 g/100 g, and an RE of 4.20%, demonstrating that the model is stable and reliable for diagnosing cotton nitrogen nutritional status.

**Figure 7:**
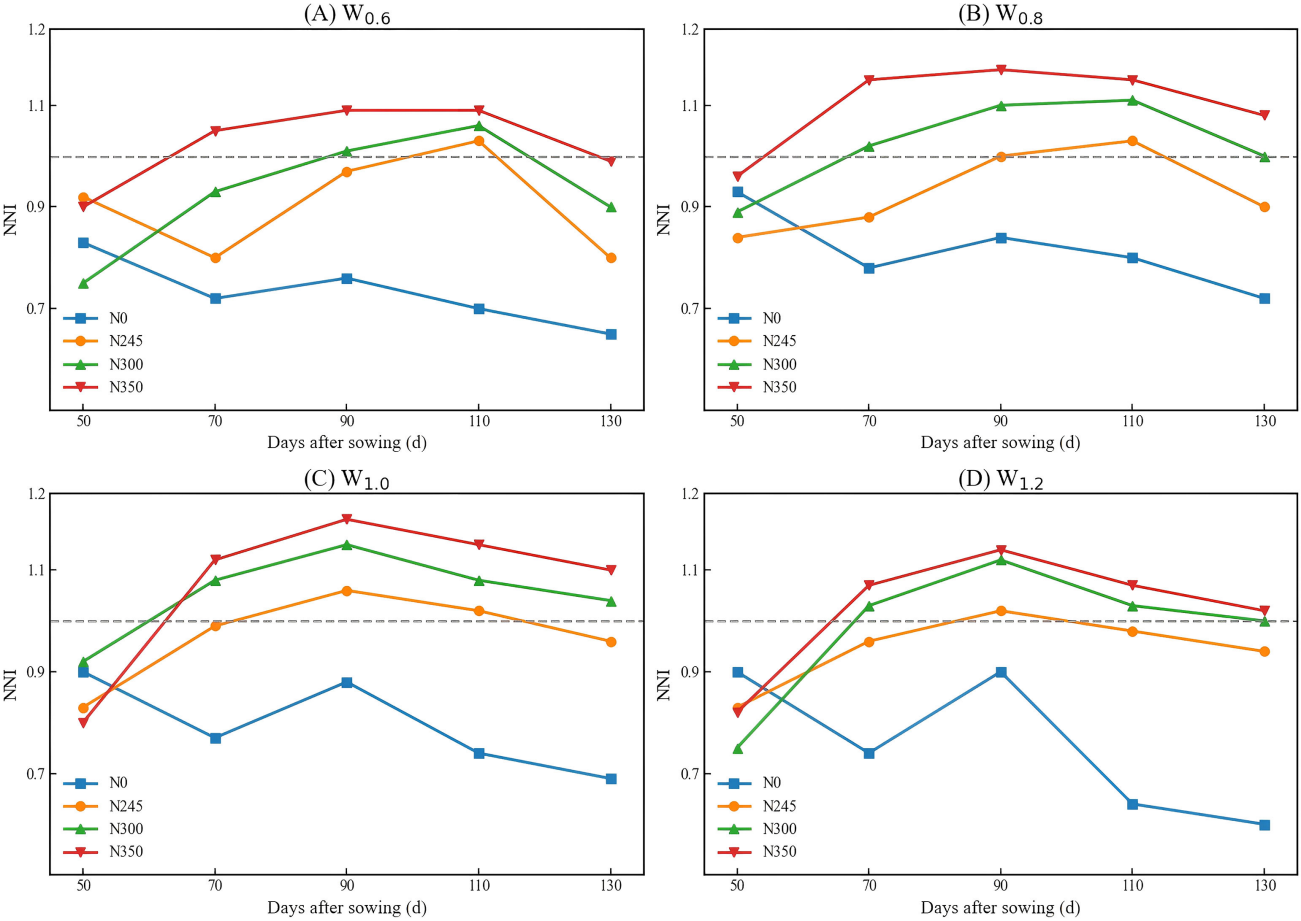
**(A–D)** represent irrigation levels of 60% ETc,80% ETc, and 100% ETc, respectively; N0, N245, N300, and N350 represent nitrogen application rates of 0, 245, 300, and 350 kg N ha^-1^, respectively.

NNI was calculated using the equation, and crop nitrogen nutritional status was evaluated accordingly. As shown in **Figure 7**, the NNI of cotton exhibited an overall pattern of increasing during 50–130 days after sowing, peaking in mid-stage, and declining thereafter. Under different irrigation conditions, NNI generally increased with increasing nitrogen application. N0 remained below 1, indicating evident nitrogen deficiency; N245 was close to 1; and N300 and N350 were mostly close to or slightly above 1, suggesting that medium to high nitrogen rates maintained adequate nitrogen nutrition. Under W0.6, NNI was generally low, indicating that drought limited nitrogen uptake. W0.8 and W1.0 exhibited the highest NNI values, suggesting that appropriate irrigation promotes nitrogen utilization, whereas W1.2 showed a slight decline in later stages, possibly due to leaching losses.

### 3.5 Analysis of the influence of INDI on NNI across different growth stages

**Figure 8:**
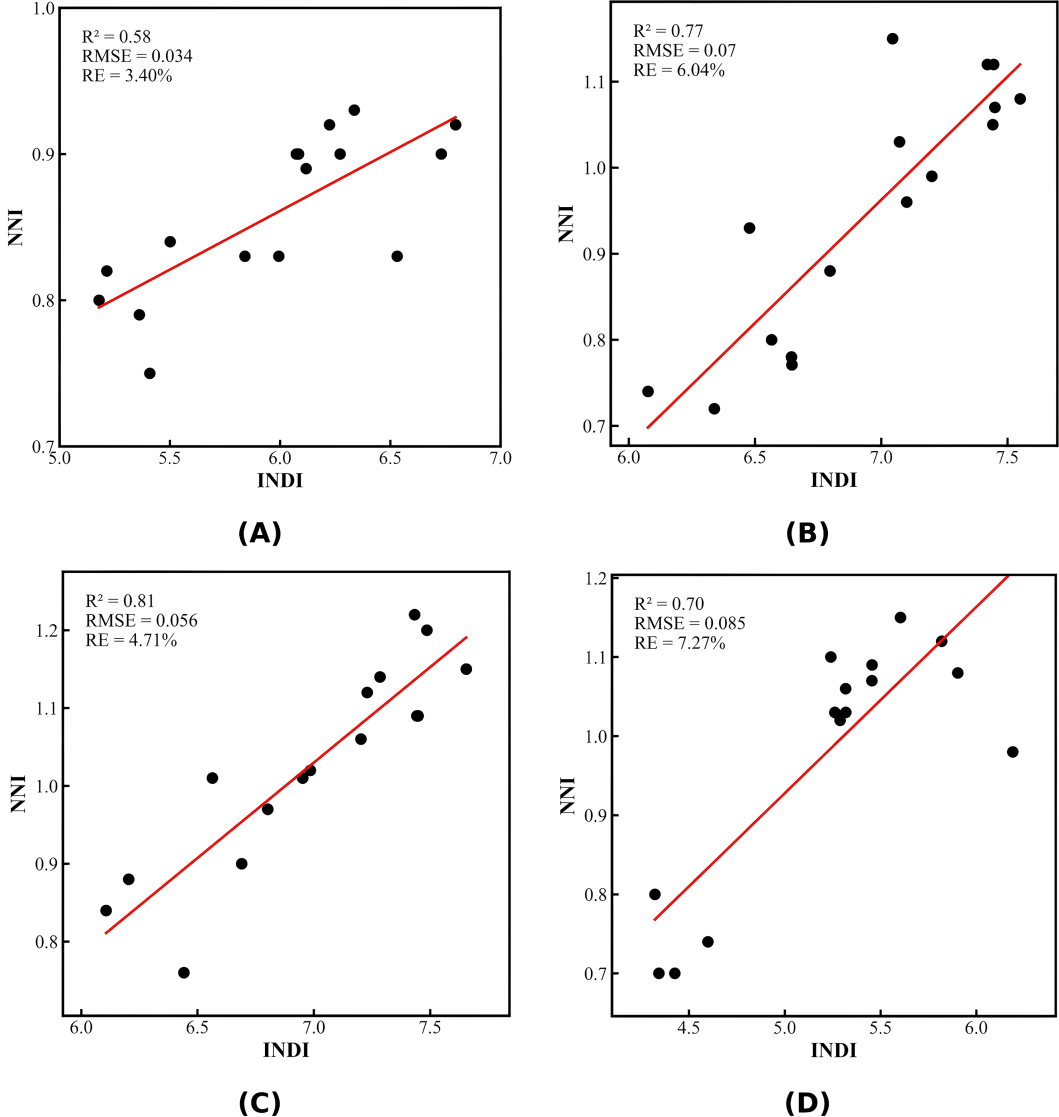
Correlation between INDI and NNI at different growth stages. **(A-D)** correspond to the squaring, flowering, boll-setting, and boll-opening stages, respectively.

To verify the capability of the integrated diagnostic index (INDI) to represent the nitrogen nutritional status of cotton, fitting analyses between INDI and the nitrogen nutrition index (NNI) were conducted at the squaring, flowering, boll-setting, and boll-opening stages, as shown in **Figure 8**. The results showed that INDI was significantly and positively correlated with NNI at all growth stages, with the strength of correlation varying across stages. At the squaring stage, the coefficient of determination between INDI and NNI was R² = 0.58, indicating a moderate correlation. As this stage corresponds to the early vegetative growth period, nitrogen accumulation is strongly affected by environmental factors, leading to relatively weaker explanatory power of INDI. During the flowering stage, the correlation between INDI and NNI increased markedly (R² = 0.77). As flowering is a critical period for nitrogen uptake, canopy structure becomes largely established, and both LAI and LNWupper exhibit higher sensitivity to nitrogen variation, enabling INDI to more reliably reflect nitrogen nutritional status. The highest correlation was observed at the boll-setting stage (R² = 0.81), during which photosynthesis reaches its peak and nitrogen allocation increasingly shifts toward reproductive organs. The integrated canopy structural characteristics and upper-leaf nitrogen attributes within INDI enable precise detection of nitrogen variation, resulting in the best inversion performance. During the boll-opening stage, the correlation between INDI and NNI declined slightly (R² = 0.70) but remained relatively high. At this stage, nitrogen is progressively transported to the bolls, and leaf function declines, reducing canopy spectral sensitivity to nitrogen. Nevertheless, the integrated INDI still maintained strong diagnostic capability.

Overall, results from the four growth stages demonstrated that INDI can consistently reflect the nitrogen nutritional status of cotton, with the strongest diagnostic performance at the boll-setting stage, followed by the boll-opening stage, a notable improvement during flowering, and relatively weaker performance during squaring. Overall, results from the four growth stages demonstrated that INDI can consistently reflect the nitrogen nutritional status of cotton, with the strongest diagnostic performance at the boll-setting stage, followed by the boll-opening stage, a notable improvement during flowering, and relatively weaker performance during squaring.

## 4 Discussion

Traditional nitrogen diagnosis methods often rely on single indicators such as SPAD, leaf nitrogen concentration (LNC), or leaf area index (LAI), but these indicators exhibit pronounced stage-specific limitations. Previous studies have demonstrated that LAI tends to saturate in dense canopies, making it difficult to distinguish differences among medium- to high-nitrogen treatments (Haboudane et al., 2004), whereas leaf nitrogen concentration is greatly influenced by the growth dilution effect, which may lead to overestimation or underestimation of nitrogen supply when comparing across the entire growth period (Lemaire and Gastal, 1997). Although SPAD is widely used in field applications, it is highly sensitive to light conditions, leaf position, and cultivar differences, resulting in poor consistency across different measurement periods (Gitelson and Merzlyak, 1994). In this study, a weighted integration of the canopy structural indicator LAI and the upper-leaf nitrogen weight LNWupper was used to construct the Integrated Nitrogen Diagnosis Index (INDI). Mechanistically, this index simultaneously represents “crop nitrogen demand (LAI)” and “actual nitrogen supply within the plant (LNWupper),” partially overcoming the limitations associated with single indicators. Compared with previous studies that developed nitrogen diagnosis models based on a single physiological or spectral indicator (Yao et al., 2020; Li et al., 2022), INDI exhibited stable nitrogen response characteristics across the squaring, flowering, boll-setting, and boll-opening stages (R² = 0.58–0.81). This demonstrates that the integration of structural information and nitrogen accumulation improves diagnostic consistency across growth stages. Li et al. (Mistele and Schmidhalter, 2008) reported in wheat that combining canopy structural indices with nitrogen-related spectral indices significantly enhances the robustness of nitrogen estimation. The present study reached similar conclusions and further suggests that integrating LAI and LNWupper into a composite index is an effective approach for establishing nitrogen diagnostic systems applicable throughout the entire growth cycle.

Multispectral analysis showed that red-edge– and near-infrared–based vegetation indices such as NDRE, MRESR, CL-RE, and MRETVI exhibited higher correlations with LNWupper and INDI than traditional NDVI and EVI. This is consistent with findings reported by Clevers & Gitelson, Delegido et al. (Gitelson et al., 1996; Delegido et al., 2011) across various crops. The red-edge region lies between strong chlorophyll absorption and strong near-infrared reflectance, making it highly sensitive to chlorophyll variations induced by nitrogen changes. It also maintains strong responsiveness under medium-to-high canopy cover conditions (Gitelson, 2005; Wu et al., 2020). Therefore, this study suggests that in drip-irrigated cotton systems characterized by complex canopy structures and pronounced water–nitrogen gradients, red-edge–based inversion should serve as a core spectral component in nitrogen retrieval models. When combined with integrated physiological indices such as INDI, nitrogen diagnostic sensitivity and stability can be further enhanced.

Regarding the comparison among machine learning models, XGBoost achieved the highest accuracy for LAI, LNWupper, and INDI inversion (INDI: R² = 0.85, RMSE = 0.61), outperforming RF and GBDT. This finding is consistent with Liu et al (Huete, 1988), who reported superior performance of XGBoost in remote estimation of nitrogen content, protein content, and yield. Compared with traditional ensemble algorithms, XGBoost achieves more efficient tree structure optimization through second-order gradient information and mitigates multicollinearity and overfitting via regularization constraints and column sampling (Chen and Guestrin, 2016). The dataset in this study exhibits typical characteristics of UAV-based experiments, namely a relatively limited sample size and strong multicollinearity among high-dimensional spectral indices. Under such data conditions, the advantages of XGBoost are fully demonstrated, indicating that XGBoost is one of the preferred algorithms for constructing high-accuracy nitrogen inversion models in practical agricultural remote-sensing applications.

INDI spatial distribution maps generated using the XGBoost model clearly revealed the spatial heterogeneity of nitrogen status under different irrigation–nitrogen combinations. High-water and high-nitrogen treatments corresponded to higher INDI values, whereas low-nitrogen or low-water treatments exhibited pronounced nitrogen-deficient patches. This trend was consistent with measured NNI values and previous findings on water–nitrogen interactions (Gastal et al., 2015b). Mistele & Schmidhalter (Mistele and Schmidhalter, 2008) noted that nitrogen spatial maps obtained from UAV or proximal sensors can effectively identify potential nitrogen-deficient areas in the field and provide spatial prescription layers for variable-rate fertilization (VRA).The results of this study further demonstrate that INDI can serve not only as a point-based nitrogen diagnostic indicator but also as a core variable for constructing decision layers in field-scale nitrogen management, supporting precision fertigation and integrated water–fertilizer regulation in drip-irrigated cotton systems.

By constructing critical nitrogen dilution curves under different irrigation levels and calculating NNI, the diagnostic performance of INDI was independently validated. INDI showed significant positive correlations with NNI across the four key growth stages, with the highest correlation in the boll-setting stage and the lowest in the squaring stage. This pattern aligns with the theory proposed by Lemaire et al. (2008) that crop nitrogen diagnosis should simultaneously consider biomass accumulation and nitrogen concentration. During early vegetative growth, environmental constraints more strongly limit nitrogen uptake, whereas during reproductive growth and boll-setting, the coupling between nitrogen supply and biomass accumulation becomes more stable. This pattern aligns with the theory proposed by Lemaire et al. (2008) that crop nitrogen diagnosis should simultaneously consider biomass accumulation and nitrogen concentration. During early vegetative growth, environmental constraints more strongly limit nitrogen uptake, whereas during reproductive growth and boll-setting, the coupling between nitrogen supply and biomass accumulation becomes more stable. Therefore, INDI can be considered highly compatible with the Nc–NNI framework for representing the balance between nitrogen supply and plant demand, and it holds strong potential as a key bridge for extending the critical nitrogen theory into spatial remote-sensing applications.

Although INDI and its remote-sensing inversion framework demonstrated good applicability and stability in this study, several limitations should be acknowledged. First, this study was conducted in drip-irrigated cotton fields in Changji, Xinjiang using a single cultivar (“Zhongmian 113”). Spectral–physiological relationships may differ across cultivars, cropping systems, and climatic regions; thus, validation across larger spatial scales, multiple years, and diverse cultivars is needed. Second, this study used a multispectral sensor with limited red-edge and near-infrared bands. Hyperspectral data offer greater potential for characterizing red-edge curve features and spectral shape parameters (Zhu et al., 2018). Thus, future work may consider multi-sensor data fusion to further improve nitrogen diagnostic accuracy and robustness. In the future, it will be necessary to develop decision rules and prescription-generation modules based on the current INDI inversion framework and to explore the combined effects of INDI-based zonal management and variable-rate fertilization strategies on yield formation, nitrogen-use efficiency, and environmental outcomes.

## 5 Conclusion

This study demonstrates that vegetation indices in the red-edge and near-infrared regions (such as NDRE and CL-RE) exhibit the strongest correlations with LNWupper and INDI, and can thus serve as core variables for cotton nitrogen inversion. The XGBoost model achieved the best performance among the three target variables and can be recommended as the optimal model for spatial inversion of INDI and nitrogen diagnosis. The INDI constructed by integrating LAI and LNWupper overcomes the limitations of single indicators, such as saturation and susceptibility to dilution effects, significantly enhancing the stability of nitrogen diagnosis across growth stages and accurately capturing changes in nitrogen status.

## Data Availability

The raw data supporting the conclusions of this article will be made available by the authors, without undue reservation.

## Supplementary material

Supplementary Figure 1 Comparison of LAI and LNWupper weights under different weighting schemes.

Supplementary Table 1 Descriptive statistics and weights of LAI and LNWupper under different weighting methods.

Supplementary Table 2 Robustness check: agreement of INDI derived from different weighting schemes.

## References

[B1] Bansal S. Gupta D. Panchal V. K. Kumar S. (2009). Swarm intelligence inspired classifiers (Berlin, Heidelberg: Springer Proceedings).

[B2] Barnes E. M. Clarke T. R. Richards S. E. Colaizzi P. D. Haberland J. Kostrzewski M. . (2000). Red edge NDVI (Denver, CO, USA: Pecora 15 Proceedings).

[B3] Bradstreet R. B. (1954). Kjeldahl method for organic nitrogen. Anal. Chem. 26, 185–187. doi: 10.1021/ac60085a028, PMID: 41799136

[B4] Chen T. Guestrin C. (2016). XGBoost (New York, NY, USA: ACM SIGKDD Proceedings), 785–794. doi: 10.1145/2939672.2939785, PMID:

[B5] Clevers J. G. P. W. Gitelson A. A. (2013). Remote estimation of crop and grass chlorophyll and nitrogen content using red-edge bands. Int. J. Appl. Earth Observ. Geoinform. 23, 344–351. doi: 10.1016/j.jag.2012.10.008, PMID: 41815951

[B6] Delegido J. Verrelst J. Alonso L. Moreno J. (2011). Evaluation of Sentinel-2 red-edge bands. IEEE JSTARS 4, 1074–1082. doi: 10.1109/JSTARS.2011.2160940, PMID: 22164004 PMC3231680

[B7] Gastal F. . (2015a). Crop physiology (London, UK: Academic Press), 161–206. doi: 10.1016/B978-0-12-417104-6.00007-3, PMID:

[B8] Gastal F. Lemaire G. Durand J. L. (2015b). Quantifying crop responses to nitrogen and water stresses in multi-resource environments. Field Crops Res. 181, 1–3.

[B9] Gitelson A. A. (1996). Red-edge NDVI. Remote Sens. Environ. 58, 289–298. doi: 10.1016/S0034-4257(96)00072-7, PMID: 41617830

[B10] Gitelson A. A. (2005). Remote estimation of canopy chlorophyll. Geophys. Res. Lett. 32, L08403. doi: 10.1029/2005GL022688, PMID: 40890438

[B11] Gitelson A. A. Kaufman Y. J. Merzlyak M. N. (1996). Use of a green channel in remote sensing. Remote Sens. Environ. 58, 289–298. doi: 10.1016/S0034-4257(96)00072-7, PMID: 41617830

[B12] Gitelson A. A. Kaufman Y. J. Merzlyak M. N. (2003a). Use of a green channel. Remote Sens. Environ. 90, 24–36.

[B13] Gitelson A. A. Gritz Y. Merzlyak M. N. (2003b). Chlorophyll–reflectance relationships. J. Plant Physiol. 160, 271–282. doi: 10.1078/0176-1617-00887, PMID: 12749084

[B14] Gitelson A. A. Merzlyak M. N. (1994). Quantitative estimation of chlorophyll-a using reflectance spectra: Experiments with autumn chestnut and maple leaves. J. Photochem. Photobiol. B: Biol. 22, 247–252. doi: 10.1016/1011-1344(93)06963-4

[B15] Gitelson A. Merzlyak M. N. (1996). Signature analysis of leaf reflectance spectra. J. Plant Physiol. 148, 494–500. doi: 10.1016/S0176-1617(96)80284-7, PMID: 41617830

[B16] Goel N. S. (1988). Vegetation canopy reflectance models. Remote Sens. Rev. 5, 1–28.

[B17] Haboudane D. Miller J. R. Pattey E. Zarco-Tejada P. J. Strachan I. B. (2004). Hyperspectral vegetation indices and novel algorithms for predicting green LAI of crop canopies. Remote Sens. Environ. 90, 337–352. doi: 10.1016/j.rse.2003.12.013, PMID: 41815951

[B18] Hou X. H. (2022). Study on water–nitrogen coupling effect. Doctoral dissertation. (Yangling, China: Northwest A&F University).

[B19] Huete A. (1988). SAVI. Remote Sens. Environ. 25, 295–309. doi: 10.1016/0034-4257(88)90106-X

[B20] Inoue Y. Sakaiya E. Zhu Y. Takahashi W. (2012a). Diagnostic mapping of canopy nitrogen content. Remote Sens. Environ. 126, 210–221. doi: 10.1016/j.rse.2012.08.026, PMID: 41815951

[B21] Inoue Y. Peñuelas J. Miyata A. Mano M. (2012b). Multi-sensor assessment of canopy phenology in rice. Remote Sens. Environ. 123, 457–466. doi: 10.1016/j.rse.2012.08.026, PMID: 41815951

[B22] Lemaire G. Gastal F. (1997). “ N uptake and distribution in plant canopies,” in Diagnosis of the Nitrogen Status in Crops (Berlin, Heidelberg: Springer), 3–43.

[B23] Lemaire G. Jeuffroy M. H. Gastal F. (2008). Diagnosis tool for plant and crop N status in vegetative stage. Eur. J. Agron. 28, 614–624. doi: 10.1016/j.eja.2008.01.005, PMID: 41815951

[B24] Lemaire G. van Oosterom E. Jeuffroy M. H. Gastal F. Justes E. (2007). Crop–soil interactions in the context of nitrogen and water management. Eur. J. Agron. 28, 414–424.

[B25] Li F. Wang Q. Zhu X. Zhu Y. Cao W. Yao X. (2022). Improving nitrogen status estimation. Field Crops Res. 283, 108533. doi: 10.1016/j.fcr.2022.108533, PMID: 41815951

[B26] Liu L. Wang J. Zhu W. Huang J. Yao X. (2021). Machine learning for wheat protein. Remote Sens. 13, 712. doi: 10.3390/rs13040712, PMID: 41725453

[B27] Mistele B. Schmidhalter U. (2008). Spectral reflectance for N status. Precis. Agric. 9, 1–13. doi: 10.1007/s11119-008-9044-0, PMID: 41816700

[B28] Qi J. Chehbouni A. Huete A. R. Kerr Y. H. Sorooshian S. (1994). Modified soil-adjusted vegetation index. Remote Sens. Environ. 48, 119–126. doi: 10.1016/0034-4257(93)90134-9

[B29] Sokal R. R. Braumann C. A. (1980). Significance tests for coefficients of variation. Syst. Zool. 29, 50–66. doi: 10.2307/2412626

[B30] Tao H. B. Lin S. (2006). Leaf area measurement methods. Plant Physiol. Commun. 42, 496–498.

[B31] Ulrich A. (1952). Physiological bases for assessing the nutritional requirements of plants. Annu. Rev. Plant Physiol. 3, 207–228. doi: 10.1146/annurev.pp.03.060152.001231, PMID: 41139587

[B32] Wu C. Cao W. Zhu Y. Yao X. Tian T. Tian Y. (2020). Improving nitrogen estimation. Field Crops Res. 247, 107583. doi: 10.1016/j.fcr.2019.107583, PMID: 41815951

[B33] Wu C. Niu Z. Tang Q. Huang W. (2008). Estimating chlorophyll content from hyperspectral vegetation indices: Modeling and validation. Agric. For. Meteorol. 148, 1230–1241. doi: 10.1016/j.agrformet.2008.03.005, PMID: 41815951

[B34] Yao X. Tang L. Cao W. Tian Y. Zhu Y. Liu X. (2020). Estimating leaf nitrogen. Precis. Agric. 21, 72–95. doi: 10.1007/s11119-019-09676-8, PMID: 41816700

[B35] Zhu Y. Cao W. Zhu Y. Yao X. Tian T. Tian Y. (2018). Red-edge position for rice N concentration. Precis. Agric. 19, 440–457. doi: 10.1007/s11119-017-9526-2, PMID: 41816700

