# Correction: Construction and UAV-based inversion of integrated nitrogen diagnosis index for cotton using multispectral imagery

**DOI:** 10.3389/fpls.2026.1850637

**Published:** 2026-04-23

**Authors:** Dongdong Zhu, Qiuping Fu, Zhenghu Ma, Shudong Lin, Qinglong Geng, Ming Hong, Jinghua Zhao, Liang Ma, Yingjie Ma, Quanjiu Wang

**Affiliations:** 1 College of Water Conservancy and Civil Engineering, Xinjiang Agricultural University, Urumqi, China; 2 Xinjiang Future Irrigation District Engineering Technology Research Center, Urumqi, China; 3 Institute of Agricultural Resources and Environment, Xinjiang Academy of Agricultural Sciences, Urumqi, China; 4 National Key Laboratory of Water Engineering Ecological Environment in Arid Areas, Xi’an University of Technology, Xi’an, China

**Keywords:** cotton, integrated nitrogen diagnosis index, machine learning, nitrogen diagnosis, nitrogen nutrition index, UAV

Affiliation 2 “Xinjiang Future Irrigation District Engineering Technology Research Center, Urumqi, China” was erroneously given as “Wensu County, Aksu, Xinjiang, China”.

Affiliation 3 “Institute of Agricultural Resources and Environment, Xinjiang Academy of Agricultural Sciences, Urumqi, China” was erroneously given as “Institute of Agricultural Resources and Environment, Xinjiang Academy of Agricultura Sciences, Urumgi, China”.

Affiliation 4 “National Key Laboratory of Water Engineering Ecological Environment in Arid Areas, Xi’an University of Technology, Xi’an, China” was erroneously given as “INational Key Laboratory of Water Engineering Ecological Environment in Arid Areas, Xi’an University of Technology, Xi’an, China”.

**Equation 12** in section “2.9 *Model construction and validation*” was erroneously given as “[ $RMSE=\frac{1}{n}\sum_{i=1}^{n}{\left({\hat{y}}_{i}-y_{i}\right)}^{2}$]”. The correct equation is “[ $RMSE=\sqrt{\frac{1}{n}\sum_{i=1}^{n}{\left({\hat{y}}_{i}-y_{i}\right)}^{2}}$]”.

The original version of this article has been updated.

