# Supplementary material for: Construction and UAV-based inversion of integrated nitrogen diagnosis index for cotton using multispectral imagery
Source: Front Plant Sci. 2026 Mar 17;17:1757798. doi: 10.3389/fpls.2026.1757798 (PMC13036123; doi:10.3389/fpls.2026.1757798)
Supplement: Supplementary Figure 1 — Comparison of LAI and LNWupper weights under different weighting schemes. [file DataSheet1.doc]

**Supplementary materials**


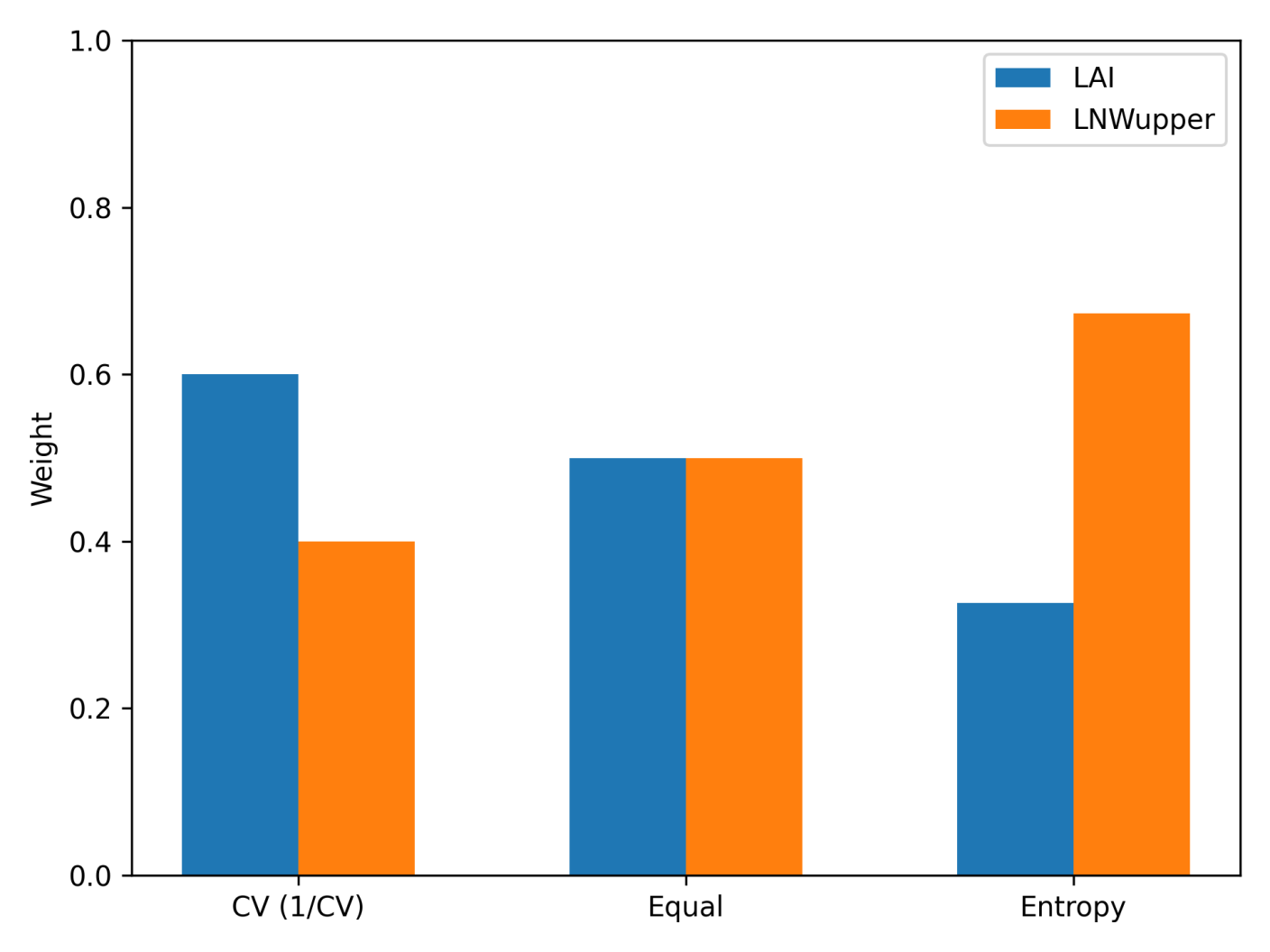


Figure S1. Comparison of LAI and LNWupper weights under different weighting schemes.

Table S1. Descriptive statistics and weights of LAI and LNWupper under different weighting methods.

| **Indicator** | **Mean** | **SD** | **CV** | **Weight_CV(1/CV)** | **Weight_equal** | **Weight_entropy** | **Entropy_e** |
| --- | --- | --- | --- | --- | --- | --- | --- |
| LAI | 4.004 | 1.342 | 0.335 | 0.600 | 0.5 | 0.327 | 0.991 |
| LNWupper | 4.471 | 2.248 | 0.503 | 0.400 | 0.5 | 0.673 | 0.982 |

Table S2. Robustness check: agreement of INDI derived from different weighting schemes.

| **Stage** | **Pair** | **Spearman_rho** |
| --- | --- | --- |
| Overall | INDI (CV weights) vs INDI (equal weights) | 0.985515175 |
| Overall | INDI (CV weights) vs INDI (entropy weights) | 0.895952687 |
| Overall | INDI (equal weights) vs INDI (entropy weights) | 0.957342673 |
| Boll opening stage | INDI (CV weights) vs INDI (equal weights) | 0.985260161 |
| Boll opening stage | INDI (CV weights) vs INDI (entropy weights) | 0.899683154 |
| Boll opening stage | INDI (equal weights) vs INDI (entropy weights) | 0.958881231 |
| Flowering stage | INDI (CV weights) vs INDI (equal weights) | 0.985971979 |
| Flowering stage | INDI (CV weights) vs INDI (entropy weights) | 0.906810882 |
| Flowering stage | INDI (equal weights) vs INDI (entropy weights) | 0.962052293 |
| Budding stage | INDI (CV weights) vs INDI (equal weights) | 0.981856456 |
| Budding stage | INDI (CV weights) vs INDI (entropy weights) | 0.871719127 |
| Budding stage | INDI (equal weights) vs INDI (entropy weights) | 0.945096269 |
| Boll-setting stage | INDI (CV weights) vs INDI (equal weights) | 0.98627754 |
| Boll-setting stage | INDI (CV weights) vs INDI (entropy weights) | 0.903220542 |
| Boll-setting stage | INDI (equal weights) vs INDI (entropy weights) | 0.959352593 |
